# Human Health Effects of Biphenyl: Key Findings and Scientific Issues

**DOI:** 10.1289/ehp.1509730

**Published:** 2015-11-03

**Authors:** Zheng Li, Karen A. Hogan, Christine Cai, Susan Rieth

**Affiliations:** National Center for Environmental Assessment, U.S. Environmental Protection Agency, Washington, DC, USA

## Abstract

**Background:**

In support of the Integrated Risk Information System (IRIS), the U.S. Environmental Protection Agency (EPA) has evaluated the human health hazards of biphenyl exposure.

**Objectives:**

We review key findings and scientific issues regarding expected human health effects of biphenyl.

**Methods:**

Scientific literature from 1926 through September 2012 was critically evaluated to identify potential human health hazards associated with biphenyl exposure. Key issues related to the carcinogenicity and noncancer health hazards of biphenyl were examined based on evidence from experimental animal bioassays and mechanistic studies.

**Discussion:**

Systematic consideration of experimental animal studies of oral biphenyl exposure took into account the variety of study designs (e.g., study sizes, exposure levels, and exposure durations) to reconcile differing reported results. The available mechanistic and toxicokinetic evidence supports the hypothesis that male rat urinary bladder tumors arise through urinary bladder calculi formation but is insufficient to hypothesize a mode of action for liver tumors in female mice. Biphenyl and its metabolites may induce genetic damage, but a role for genotoxicity in biphenyl-induced carcinogenicity has not been established.

**Conclusions:**

The available health effects data for biphenyl provides suggestive evidence for carcinogenicity in humans, based on increased incidences of male rat urinary bladder tumors at high exposure levels and on female mouse liver tumors. Kidney toxicity is also a potential human health hazard of biphenyl exposure.

**Citation:**

Li Z, Hogan KA, Cai C, Rieth S. 2016. Human health effects of biphenyl: key findings and scientific issues. Environ Health Perspect 124:703–712; http://dx.doi.org/10.1289/ehp.1509730

## Introduction

Biphenyl exists naturally as a component of crude oil and coal tar. Biphenyl is currently used as a chemical synthesis intermediate (including in the synthesis of the sodium salt of 2-hydroxybiphenyl, a pesticide known as Dowicide 1), as a dye carrier in polyester dyeing, and as a component in heat transfer fluids (consisting of 26.5% biphenyl and 73.5% diphenyl oxide). Biphenyl has been used as a fungistat, most commonly to preserve packaged citrus fruits or in plant disease control (as reviewed by HSDB 2014; IPCS 1999); U.S. registration of biphenyl as a pesticide (fungistat and antimicrobial agent) was cancelled [U.S. Environmental Protection Agency (EPA) 1998].

Biphenyl is listed as a high-volume production (HPV) chemical in the United States (Kim et al. 2015) and in Europe (OECD 2004). An HPV chemical is produced or imported into the United States in quantities of 1 million pounds per year (Bishop et al. 2012). Biphenyl is also listed as a hazardous air pollutant under the Clean Air Act (U.S. EPA 2014b, 2016b), and has been found at multiple Superfund sites (U.S. EPA 2014a).

To support programmatic needs, the U.S. EPA’s Integrated Risk Information System (IRIS) Program developed an updated human health assessment of biphenyl (U.S. EPA 2013). Overall, the toxicological review was developed according to the general framework for risk assessment set forth by the National Research Council (NRC 1983, 1994), as well as applicable U.S. EPA guidance (U.S. EPA 2016a). The literature on relevant health effects for biphenyl was identified through comprehensive searches of online scientific databases [including PubMed, Toxline, Biosis, Embase, Toxcenter, Current Contents, and Toxic Substances Control Act Test Submissions (TSCATS)] using the Chemical Abstracts Service Registry Number (CASRN; 92-52-4), the common chemical name, and synonyms. Primary peer-reviewed literature published from 1926 through September 2012 was included in the evaluation of the health hazards of biphenyl exposure. Public submissions to the U.S. EPA were also considered for inclusion. The Toxicological Review of Biphenyl (U.S. EPA 2013) provides additional details regarding the literature search strategy, all pertinent evidence and its interpretation in light of relevant U.S. EPA guidance, and quantitative analyses of carcinogenicity and effects other than cancer associated with oral exposure to biphenyl, including dose–response analyses and toxicity value derivations. Completion of the Toxicological Review of Biphenyl involved review by scientists within the U.S. EPA and other federal agencies and by the Executive Office of the President. In addition, the U.S. EPA elicited public comment and an external peer review, which was held at a public meeting.

In this article, we continue a series of recent IRIS assessments (Chiu et al. 2013; Guyton et al. 2014; Schlosser et al. 2015) by highlighting the following key scientific issues encountered in evaluating the potential impact of biphenyl exposure on human health: biphenyl metabolism; the carcinogenicity of biphenyl, based on the analyses of multiple laboratory animal bioassays and mechanistic data; and the noncancer effects associated with biphenyl exposure, with a focus on renal toxicity.

## Metabolism

Evidence for biphenyl metabolism in humans comes from a small number of *in vitro* studies using tissues of human origin. In the human liver, biphenyl is hydroxylated to the following hydroxybiphenyls (HBPs), in order of increasing amounts (based on measures of hydroxylation activities): 2-HBP < 3-HBP < 4-HBP (Benford et al. 1981). Powis et al. (1987) showed that 4-HBP is conjugated with glucuronic acid and sulphate in human liver and kidney tissue slices. Sulphotransferase activity for 2-, 3-, and 4-HBP was detected in various human surgical tissue sample preparations (liver, intestinal mucosa, lung, kidney, bladder, and brain) incubated with one of the hydroxybiphenyl isomers, with the highest activity found in the liver (Pacifici et al. 1991).

Human metabolism of biphenyl appears to be qualitatively similar to metabolism in experimental animals. In laboratory animals, biphenyl is converted to a range of hydroxylated metabolites (Halpaap-Wood et al. 1981a, 1981b; Meyer et al. 1976a, 1976b; Meyer and Scheline 1976; Meyer 1977). These metabolites have been detected in urine as compounds conjugated to glucuronic acid and sulphate. 4-HBP is a major metabolite in the rat, mouse, guinea pig, rabbit, and pig (Halpaap-Wood et al. 1981b; Meyer and Scheline 1976; Meyer 1977). Similar metabolites have been identified in the various mammalian species tested, although quantitative differences in metabolite formation across species are evident. For example, 4,4´-dihydroxybiphenyl was identified as a major metabolite in the rat (Halpaap-Wood et al. 1981a; Meyer and Scheline 1976; Meyer 1977), whereas 3,4´-dihydroxybiphenyl was identified as a major urinary metabolite in two strains of mice (Halpaap-Wood et al. 1981a). 2-HBP is a minor pathway in both rats and mice, but is more easily detected in mice than in rats (Halpaap-Wood et al. 1981a, 1981b).

*In vitro* studies using animal systems support the findings from *in vivo* urinary metabolite investigations that a range of hydroxylated biphenyl metabolites are formed and that 4-HBP is a major metabolite. Isolated rat and hamster hepatocytes metabolized biphenyl primarily to 4-HBP and also to 4,4´-dihydroxybiphenyl (Bianco et al. 1979; Wiebkin et al. 1976, 1984). Billings and McMahon (1978) reported that 2- and 3-HBP were detected (at similar levels) in mouse microsomes; whereas almost all hydroxylation of biphenyl in rat microsomes gave rise to 4-HBP, supporting the *in vivo* finding that more 2-HBP is formed in mice than in rats.

Biphenyl metabolism is mediated by cytochrome (CYP) monooxygenases. Evidence of an arene oxide intermediate was reported by Billings and McMahon (1978). Studies in rodents to investigate the induction of Phase I enzymes that catalyze biphenyl hydroxylation demonstrated that multiple CYP enzymes (e.g., CYP1A2 and CYP3A4) are likely involved in biphenyl hydroxylation. For example, pretreatment of rats (Wistar and CD strains) or mice (ICI and C57BL/6JHan strains) with phenobarbital (an inducer of CYP3A4, 2B6, and 2C8) or 3-methylcholanthrene (an inducer of CYP1A2) increased activities of liver microsomes to produce 2-, 3-, or 4-HBP from biphenyl to varying degrees depending on the inducer (Creaven and Parke 1966; Haugen 1981; Stuehmeier et al. 1982). β-Naphthoflavone (an inducer of CYP1A2; also known as 5,6-benzoflavone) enhanced the urinary excretion of multiple hydroxylated biphenyl metabolites in male Sprague-Dawley rats, and shifted the principal metabolite from 4-HBP to 2-HBP and 2,5-dihydroxybiphenyl in male C57BL/6Tex mice (Halpaap-Wood et al. 1981a). CYP enzymes catalyzing hydroxylation of biphenyl and enzymes catalyzing the conjugation of hydroxybiphenyls with glucuronic acid or sulphate have been detected in a number of mammalian tissues, with the highest levels found in the liver (Parkinson and Ogilvie 2008).

## Carcinogenicity

The carcinogenic potential of biphenyl has not been investigated in humans. In 2-year animal bioassays, biphenyl dietary exposure was associated with an increased incidence of urinary bladder tumors in male F344 rats (Umeda et al. 2002) and liver tumors in female BDF_1_ mice (Umeda et al. 2005). Although earlier dietary studies in rats and mice did not provide clear evidence of carcinogenicity (Ambrose et al. 1960; Dow Chemical 1953; Imai et al. 1983; NCI 1968; Shiraiwa et al. 1989), these earlier studies differed in a number of respects (e.g., study design, conduct, and/or reporting of results; see Table 1) and were considered less informative for evaluating the carcinogenicity of biphenyl than were the studies conducted by Umeda et al. (2002) and Umeda et al. (2005). Summaries of the bioassays of biphenyl in rats and mice, including key study design features, are provided in Table 1. The following sections integrate key aspects of the animal carcinogenicity evidence with genotoxicity and other mechanistic evidence in order to evaluate the potential for human carcinogencity.

**Table 1:** Summary of tumor studies and related end points in biphenyl bioassays.

Reference	Strain/group size Duration Route/dose (mg/kg-day)	Tumor findings	Comments
Rat studies
Umeda et al. 2002	F344/50/sex/group 24 months Diet/M: 0, 36.4, 110, 378; F: 0, 42.7, 128, 438^*a*^	M (high-dose)—Bladder tumors (papilloma or carcinoma). Incidences (refer to Table 2) F—No treatment-related tumors	Other bladder findings: M (high-dose group only)—calculi and transitional cell hyperplasia and hematuria F (high-dose group only)—calculi and hyperplasia at lower incidence than M The physico-chemical characteristics of calculi in M and F rats differed
Shiraiwa et al. 1989	Wistar/50/sex/group 17 months Diet/M: 0, 165, 353; F: 0, 178, 370^*b*^	M & F—No treatment-related tumors	Bladder findings: both M and F developed hematuria; urinary bladder stones (lower incidence in F); hyperplasia and papillomatosis of the mucosa of the urinary bladder
Ambrose et al. 1960	Albino (weanling)/15/sex/group 24 months Diet/0, 1, 4, 8, 42, 84, 420, 840^*c*^	M—bladder tumors in 6 of 8 dose groups (controls–2/9; 1–2/8; 8–1/9; 84–1/9; 420–1/2; and 840–1/2) and female control rats (1/9) F—bladder tumor in control only (1/9)	Group sizes too small to establish whether bladder tumor incidence in M rats was dose related Survival in two highest dose groups only 13–33% Histopathological examination limited to terminal sacrifice animals only (*n* = 2–13 animals/group)
Pecchiai and Saffiotti 1957	Albino (M only)/8/group 13 months (with 2-month interim sacrifices) Diet/0, 250, 450^*d*^	M—tumors of the forestomach epithelium (2 papillomas, 1 squamous cell carcinoma); dose groups for animals with tumors not specified	At sacrifice, major tissues examined; bladder not included
Dow Chemical 1953	Sprague-Dawley/12/sex/group 24 months Diet/0, 7, 73, 732^*e*^	M & F—No treatment-related tumors	Outbreak of pneumonia during study Poor survival (e.g., all control M died by 18 months and only 2 of 12 mid-dose F survived to 21 months)
Mouse studies
Umeda et al. 2005	BDF_1_/50/sex/group 24 months Diet/M: 0, 97, 291, 1,050; F: 0, 134, 414, 1,420^*f*^	M—Incidences (refer to Table 3) of hepatocellular adenoma or carcinoma (combined) statistically significantly decreased with increasing dose F—Incidences (refer to Table 3) of hepatocellular adenomas or carcinomas statistically significantly increased with increasing dose	M: Although decreased, the incidences of hepatocellular adenoma or carcinoma (combined) were within the range of historical controls for that laboratory F: The incidences of hepatocellular tumors (adenomas, carcinomas, and adenomas and carcinomas combined) in mid- and high-dose F mice exceeded the range of historical controls for that laboratory
Imai et al. 1983	ddY (F only)/60/group 24 months Diet/0, 855^*g*^	No treatment-related tumors	Histopathological findings reported only for 34–37 mice/group
Innes et al. 1969; NCI 1968	2 strains of F1 hybrids: B6C3F_1_ (C57BL/6 × C3H/Anf) and B6AKF_1_ (C57BL/6 × AKR)/18/sex/ strain/group 18 months: Through week 3: 0, 215 (via gavage) Remainder of study: 0, 91 (via diet)^*h*^	Reticular cell sarcoma incidence significantly elevated in B6AKF_1_ F mice (controls-4/82, treated-4/17), but not in M mice (controls-1/90, treated-0/17) of this strain or B6C3F_1_ mice of either sex (M: controls-5/79, treated-1/17; F: controls-4/87, treated-0/18)	Histopathological examination limited to total chest contents, liver, spleen, kidneys with adrenals, stomach, and genital organs Biological significance of reticular cell sarcoma is not clear because the origin of this tumor was not specified. The tumor response may have been influenced by early-life exposure in this study, starting at 1 week of age
Abbreviations: M, male; F, female. ^***a***^Doses calculated from dietary concentrations (0, 500, 1,500, or 4,500 ppm), time-weighted average body weights estimated from the graphically depicted data (Umeda et al. 2002; Figure 1), and chronic reference values for food consumption in F344 rats (U.S. EPA 1988). ^***b***^Doses calculated based on reported values for mean daily biphenyl intake (mg biphenyl/rat) and mean initial and final body weights for each study group. Concentrations in feed were 0, 2,500, or 5,000 ppm. ^***c***^Doses calculated from dietary concentrations (0, 10, 50, 100, 500, 1,000, 5,000, or 10,000 ppm) and from U.S. EPA (1988) reference values for body weight and food consumption in F344 rats (averages of values for M and F). ^***d***^Doses estimated by authors. ^***e***^Doses calculated from dietary concentrations (0, 100, 1,000, or 10,000 ppm) and from U.S. EPA (1988) chronic reference values for body weight and food consumption in Sprague-Dawley rats (average values for combined sexes). ^***f***^Doses reported by authors. Concentrations in feed were 0, 667, 2,000, or 6,000 ppm. ^***g***^Doses calculated from dietary concentrations (0 or 5,000 ppm), estimated food consumption rates (U.S. EPA 1988), and reported average terminal body weight (0.037 kg). ^***h***^Doses calculated from dietary concentrations (0 or 517 ppm) and from U.S. EPA (1988) chronic reference values for body weight and food consumption in B6C3F_1_ mice (average values for combined sexes).			

### Genotoxicity

Biphenyl genotoxicity was tested in prokaryotic and eukaryotic systems and nonhuman mammalian studies, both *in vitro* and *in vivo*. Mutation and DNA repair assays in prokaryotic organisms provide consistently negative results both with and without the addition of a mammalian metabolic activation system (S9 mix, rat liver microsomal fraction) after biphenyl treatment (Bos et al. 1988; Brams et al. 1987; Chung and Adris 2003; Cline and McMahon 1977; Garrett et al. 1986; Glatt et al. 1992; Haworth et al. 1983; Hellmér and Bolcsfoldi 1992; Houk et al. 1989; Ishidate et al. 1984; Kojima and Hiraga 1978; Narbonne et al. 1987; Pagano et al. 1983, 1988; Probst et al. 1981; Purchase et al. 1978; Westinghouse Electric 1977). In a non-mammalian eukaryotic organism (*Saccharomyces cerevisiae* D7), biphenyl induced mitotic recombination and gene conversion both with and without S9 (Pagano et al. 1988).

Results of genotoxicity assays of biphenyl-exposed cultured mammalian cells were mostly negative when S9 mix was not present. For example, no mutations were detected in the hypoxanthine-guanine phosphoribosyltransferase (*Hprt*) gene in Chinese hamster V79 cells (Glatt et al. 1992) and the thymidine kinase (*tk*) locus in mouse L5178Y/TK+/– lymphoma cells (Wangenheim and Bolcsfoldi 1988) in the absence of S9. Chromosomal aberrations in several test systems were not increased significantly: Chinese hamster lung cells (Abe and Sasaki 1977; Ishidate and Odashima 1977; Ishidate et al. 1984; Sofuni et al. 1985), DNA strand breaks in mouse lymphoma L5178Y cells (Garberg et al. 1988), DNA repair in human lung fibroblasts (Snyder and Matheson 1985), and unscheduled DNA synthesis in rat primary hepatocytes (Brouns et al. 1979; Hsia et al. 1983; Probst et al. 1981; Williams et al. 1989). The only positive findings without S9 were reported by Rencüzoğullari et al. (2008) who found dose-related increases of micronuclei, chromosomal aberrations, and sister chromosomal exchanges (SCEs) in human primary peripheral lymphocytes; dose-dependent cytotoxicity was also observed.

Very few studies examined genotoxicity end points in the presence of S9, and these have reported mixed results. *Hprt* gene mutations in Chinese hamster V79 cells (Glatt et al. 1992), chromosomal aberrations in Chinese hamster lung fibroblasts (Sofuni et al. 1985), and DNA strand breaks in mouse lymphoma L5178Y cells (Garberg et al. 1988) were observed with S9. Forward mutations in mouse L5178Y/TK^+/–^ lymphoma cells were reported, but the observation of mutations was accompanied by cytotoxicity (Wangenheim and Bolcsfoldi 1988). A cell transformation assay in human and hamster cells was negative in the presence of S9 (Purchase et al. 1978).

Evaluations of the potential genotoxicity of biphenyl *in vivo* found no evidence of chromosomal aberrations in rat bone marrow following inhalation exposure to biphenyl dust (Johnston et al. 1976) or micronuclei in mouse bone marrow after a single gavage dose (Gollapudi et al. 2007). However, positive results were reported for DNA strand breaks in stomach, blood, liver, bone marrow, kidney, bladder, lung, and brain of mice administered single doses of 2,000 mg biphenyl/kg (Sasaki et al. 1997, 2002).

Genotoxicity assays of biphenyl metabolites suggest that the metabolites may be more genotoxic than the parent compound. Metabolism of 2-HBP may induce oxidative DNA damage resulting from redox cycling between 2,5-dihydroxybiphenyl and phenylbenzoquinone (Balakrishnan et al. 2002; Kwok et al. 1999). Limited evidence for this can be found in positive results in two bacterial strains developed to be sensitive to oxidative DNA damage (Fujita et al. 1985; Tani et al. 2007). Micronuclei (Balakrishnan et al. 2002) and DNA strand breaks (Sasaki et al. 1997, 2002) were reported in *in vivo* studies on 2-HBP. Whether DNA damage is caused by a direct reaction with DNA or by indirect damage from reactive oxygen species generated from redox cycling, or some combination of these mechanisms, is unknown. Studies that investigated DNA adduct formation using 2-HBP were mostly negative (Kwok et al. 1999; Smith et al. 1998), except for one study of very high doses applied to skin (Pathak and Roy 1993). One study that directly tested the mutagenicity of the major metabolite, 4-hydroxybiphenyl, in the Ames assay was positive (Narbonne et al. 1987), but no other investigations of this metabolite were located.

In summary, biphenyl and its metabolites have some ability to induce genetic damage. A more detailed review of the genotoxic potential of biphenyl and its metabolites is provided in the Toxicological Review of Biphenyl (U.S. EPA 2013).

### Urinary Bladder Tumors and Mechanistic Data Analysis

A dose-related increase in transitional cell papilloma and carcinoma of the urinary bladder was reported in male F344 rats exposed to biphenyl in the diet for 2 years (Umeda et al. 2002) (Table 2). Related bioassay findings and physiological and mechanistic data support a hypothesized mode of action for biphenyl-induced urinary bladder tumors that includes the following key events: formation of urinary bladder calculi, damage of transitional epithelial cells of the urinary bladder leading to sustained regenerative cell proliferation and hyperplasia, and promotion of initiated cells in the urinary bladder with progression to urinary bladder papillomas and carcinomas. The evidence that supports this hypothesized mode of action is discussed in the remainder of this section.

**Table 2:** Incidences of urinary bladder lesions in male and female F344 rats exposed to biphenyl in the diet for 2 years.

Exposure information	Males				Females			
Dietary concentration (ppm)	0	500	1,500	4,500	0	500	1,500	4,500
Calculated dose (mg/kg-day)^*a*^	0	36.4	110	378	0	42.7	128	438
Urinary bladder lesion
Calculi	0/50	0/50	0/50	43/50**	0/50	0/50	0/50	8/50**
Transitional cell hyperplasia
indentedtwice">Simple hyperplasia^*b*^	0/50	0/50	0/50	12/50*	0/50	0/50	1/50	1/50
indentedtwice">Nodular hyperplasia^*b*^	0/50	0/50	0/50	40/50*	1/50	0/50	0/50	5/50
indentedtwice">Papillary hyperplasia^*b*^	0/50	0/50	0/50	17/50*	0/50	0/50	0/50	4/50
Combined hyperplasia	0/50	0/50	0/50	45/50**	1/50	0/50	1/50	10/50**
Inflammatory polyp^*b*^	0/50	0/50	0/50	10/50*	0/50	0/50	0/50	0/50
Transitional cell tumors
Papilloma	0/50	0/50	0/50	10/50*	0/50	0/50	0/50	0/50
Carcinoma	0/50	0/50	0/50	24/50*	0/50	0/50	0/50	0/50
Papilloma or carcinoma (combined)	0/50	0/50	0/50	31/50**	0/50	0/50	0/50	0/50
^***a***^Calculated doses based on time-weighted average body weights and chronic reference food consumption values for F344 rats (U.S. EPA 1988). ^***b***^The incidence is the sum of animals with severity grades of slight, moderate, marked, or severe. Adapted from Umeda Y, Arito H, Kano H, Ohnishi M, Matsumoto M, Nagano K, Yamamoto S, and Matsushima T. 2002. Two-year study of carcinogenicity and chronic toxicity of biphenyl in rats. J Occup Health 44:176–183. *Statistically significant (Fisher’s exact test, *p* < 0.05) compared with untreated controls, as reported by study authors; **Statistically significant (Fisher’s exact test, *p* < 0.05) compared with untreated controls, as determined by EPA.								

Umeda et al. (2002) observed a strong association between bladder lesions, including calculi formation, transitional cell hyperplasia and papillomas/carcinomas, in male F344 rats. Urinary bladder calculi (86%) and transitional cell hyperplasia (90%) occurred at the same dietary concentration of biphenyl (4,500 ppm) that induced urinary bladder transitional cell tumors (62%) (Umeda et al. 2002). Urinary bladder calculi were present in 42 of 45 male rats with urinary bladder transitional cell hyperplasia, in 8 of 10 male rats with papillomas, and in all male rats with carcinomas. Urinary bladder calculi and transitional cell hyperplasia were not present at lower dietary concentrations, nor were tumors.

Histopathological observations of transitional cell hyperplasia in male rats exposed to biphenyl are consistent with a regenerative response to sustained mechanical irritation of the epithelium (i.e., by calculi) rather than a direct effect of biphenyl. Umeda et al. (2002) reported that the hyperplastic lesions of the bladder were focal rather than diffuse. Among all three types of hyperplasia identified in the urinary bladder of high-dose F344 male rats, nodular hyperplasia (80%) occurred more frequently than simple (24%) or papillary hyperplasia (34%) (Table 2). By contrast, there is evidence that genotoxic bladder carcinogens without calculi formation induce a different profile and distribution of hyperplasias. For example, 2-acetylaminofluorene induced a 100% incidence of diffuse, simple hyperplasia in mice treated for 13 weeks, and a lower incidence (25%) of focal, nodular hyperplasia (Frith and Rule 1978). Observations of hematuria in male F344 rats in the Umeda et al. (2002) study also suggest that damage to the transitional epithelium was causally associated with mechanical irritation caused by calculi. Specifically, Umeda et al. (2002) reported that 94% of the 32 male rats with hematuria had calculi in the bladder or kidneys.

Evidence from multiple studies of a temporal relationship among the key events further supports the hypothesized mode of action. Among male rats exposed to 4,500 ppm dietary biphenyl, urinary bladder calculi were observed in the first male rat that died (week 36), evidence of blood in the urine was observed by week 40, and incidences of bladder calculi and bloody urine that paralleled increases in mortality due to tumor formation were observed throughout the remainder of the study (Umeda et al. 2002). All but one urinary bladder tumor were identified after 75 weeks. These events provide evidence of a progression from calculi formation, to damage to the transitional epithelium, to bladder tumor induction. In a 75-week dietary study, male rats exposed to 5,000 ppm showed hematuria starting at 16 weeks, becoming more noticeable after 60 weeks of exposure (Shiraiwa et al. 1989). At the end of the 75-week treatment, calculi, hyperplasia, and papillomatosis of the mucusa in the urinary bladder, but not tumors, were observed. The late stage appearance of bladder tumors in the 2-year study by Umeda et al. (2002) suggests that the mechanical damage to the urinary bladder epithelium by calculi, evidenced by hematuria, was not sustained for sufficiently long to induce urinary bladder tumors in the 75-week Shiraiwa et al. (1989) study. Shibata et al. (1989b) observed biphenyl-induced calculi formation (microcalculi in the urine) and an increase in BrdU (5-bromo-2-deoxyuridine) incorporation (an index of urinary bladder transitional cell proliferation) more than four times higher than control in male F344 rats after 4–8 weeks exposure to 5,000 ppm biphenyl in the diet.

Although female F344 rats in the Umeda et al. (2002) study showed urinary bladder calculi (16%) and transitional cell hyperplasia (20%) at 4,500 ppm biphenyl in the diet, at a lower incidence than in male rats, urinary bladder transitional cell papillomas or carcinomas were not observed in female rats at any dose level (Table 2). The absence of female urinary bladder tumors in the presence of calculi may be explained by metabolic and physiological differences between male and female rats. The lower incidences of calculi formation in female rats are consistent with differences in biphenyl metabolism (Ohnishi et al. 2000b) and urine physiological properties (e.g., pH level) (Van Vleet et al. 2007) between male and female rats. The absence of urinary bladder tumors in female rats with urinary bladder calculi may be related to sex-specific differences in the physical properties of biphenyl-induced calculi. The potassium salt of the sulphate conjugate of 4-HBP plays a pivotal role in forming the calculi, as it is the least soluble potassium salt among conjugates formed by the biphenyl metabolites (Ohnishi et al. 2000b). Indeed, the calculi in male rats were composed primarily of potassium 4-hydroxybiphenyl-*O*-sulphate (4-HBPOSK). By contrast, the calculi in female rats were composed primarily of 4-HBP and potassium bisulphate, hydrolysis products of potassium 4-HBPOSK (Ohnishi et al. 2000b; Umeda et al. 2002).

The formation of calculi appears to result from the precipitation of the potassium salt of the sulphate conjugate of 4-HBP under the elevated pH conditions in male rat urine. Urine pH of male rats (pH = 7.97) in the 4,500-ppm group at the final week of exposure was statistically significantly higher compared with controls (pH = 7.66); whereas the urine pH of female rats exposed to 4,500 ppm for 2 years (pH = 7.26) was similar to controls (pH = 7.29) (Umeda et al. 2002). In a subchronic study, urine crystals were found only in male rats coadministered biphenyl and potassium bicarbonate (KHCO_3_) in the diet for 13 weeks; urine crystals were not observed in male rats fed biphenyl, biphenyl and potassium chloride (KCl), or biphenyl and sodium bicarbonate (NaHCO_3_) (Ohnishi et al. 2000a), suggesting both elevated urinary pH (resulting from dietary administration of KHCO_3_) and potassium were required for precipitation of crystals in urine. The sex-specific difference in pH induced by biphenyl treatment may further affect the activity of sulphatases in the kidney, as lysosomal sulphatases have optimal activity under acidic pH conditions (Hanson et al. 2004). The lower pH of female urine thus may increase sulphatase activity and facilitate the hydrolysis of biphenyl sulfate conjugates, thereby reducing calculi formation.

The physical properties and structure of calculi in male and female rats exhibited differences (Ohnishi et al. 2000b; Umeda et al. 2002) that could account for the difference in bladder tumor response in males and females. Urinary bladder calculi in male rats are highly heterogeneous; the aggregates can be triangular, pyramidal, or cubical in shape, and 0.3–1.0 cm in size, with diverse colors of white, yellow, brown, gray, and black. By contrast, calculi in female rats are of homogeneous size and spheroidal in shape, and consistently white or yellow in color. Structurally, male calculi were made of layers of 4-HBPOSK compactly covered with calcium phosphate; female calculi were needle-shaped crystals with places of open holes. Ohnishi et al. (2000b) suggested that calculus formation in males may involve a series of successive and irreversible reactions, whereas calculus formation in females appears to result from reversible reactions, including 4-HBPOSK hydrolysis. The calculi formed in male rats are more stable than those in females. Presumably calculi formed in male rats produce sufficient mechanical damage to the urinary bladder epithelium to induce tumors, whereas the calculi in female rats, with different chemical composition and physical properties, do not. More research is needed to identify the specific physical or chemical properties of biphenyl-induced calculi that are associated with mechanical damage and subsequent tumor development.

Unlike the rat, urinary bladder calculi and tumors have not been observed in mice exposed to biphenyl (Table 1). The differences in urinary bladder response between rats and mice following biphenyl dietary exposure may be explained by the differences in biphenyl metabolism in these two species—in particular to the differences in the quantities formed of the biphenyl metabolites 4-HBP (relatively greater in rats) and 2-HBP (relatively greater in mice) (see “Metabolism” section). Whether these species differences in biphenyl metabolism account for or contribute to the lack of urinary bladder calculi formation in mice is unknown (Umeda et al. 2005). Thus, species differences in bladder tumor induction requires further investigation.

As discussed previously, biphenyl and its metabolites may induce genetic damage; however, specific evidence is not available to establish a role for genotoxicity in the mode of action of biphenyl-induced urinary bladder tumors.

### Liver Tumors and Mechanistic Data Analysis

Exposure to biphenyl in the diet for 2 years was associated with a statistically significant dose-related increase in the incidence of liver tumors (hepatocellular adenomas or carcinomas) in female BDF_1_ mice (Umeda et al. 2005) (Table 3). In the same study, the incidence of liver tumors in male mice showed a statistically significant decrease; however the incidences were within the range of historical controls for this laboratory (Table 3) and may reflect the higher background rate of hepatocellular tumors in male mice relative to female mice (32% in males, 6% in females) and the dose-related decrease in body weight (about a 30% weight reduction in the highest dose group). Similar decreasing trends in liver tumors that were associated with decreased body weight in B6C3F1 mice, as also occurred in the BDF1 mice exposed to biphenyl, have been judged not to demonstrate anticarcinogenicity (e.g., Haseman and Johnson 1996; Leakey et al. 2003). Despite a similar body weight decrease (about a 25% decrease in the highest dose group), female mice still showed clear increases in tumors with increasing exposure, greater than in historical controls (Table 3).

**Table 3:** Incidences of liver tumors in male and female BDF_1_ mice exposed to biphenyl in the diet for 2 years.

Exposure information	Males				Females			
Dietary concentration (ppm)	0	667	2,000	6,000	0	667	2,000	6,000
Mean terminal body weight (g) ± SE	46.9 ± 4.9	43.1 ± 7.9	42.9 ± 6.0	32.4 ± 3.6	34.0 ± 4.0	32.5 ± 3.3	30.5 ± 3.1	25.5 ± 3.0
Calculated dose (mg/kg-day)^*a*^	0	97	291	1,050	0	134	414	1,420
Tumor^*b*^
Hepatocellular adenoma	8/50	6/49	7/50	3/50	2/50	3/50	12/50	10/49
Hepatocellular carcinoma	8/50	8/49	5/50	4/50	1/50	5/50	7/50	5/49
Hepatocellular adenoma or carcinoma (combined)	16/50*	12/49	9/50	7/50	3/50*	8/50	16/50	14/49
^***a***^Calculated doses based on time-weighted average body weights and chronic reference food consumption values for BDF_1_ mice (U.S. EPA 1988). ^***b***^Historical control data for hepatocellular tumors: male BDF_1_ mouse: adenoma—17.2% (4–34%), carcinoma—18.8% (2–42%), adenoma/carcinoma—32.2% (10–68%); female BDF_1_ mouse: adenoma—4.8% (0–10%), carcinoma—2.5% (0–8%), adenoma/carcinoma—7.1% (2–14%). Source: historical control data provided by study investigators. Adapted from Umeda et al.(JVMS, Vol.67, No.4, 2005). *Statistically significant trend (two-sided Cochran–Armitage trend test, *p* < 0.05), as determined by the U.S. EPA.								

There was no liver tumor response in either sex of B6C3F_1_ or B6AKF_1_ mice (NCI 1968), but these evaluations were carried out at lower doses than those used by Umeda et al. (2005), for a shorter duration (18 rather than 24 months), and with treatment groups of no more than 18 animals. There was no observed liver tumor response in female ddY mice (Imai et al. 1983)—males were not tested—with exposure at a level intermediate to the two higher exposures evaluated by Umeda et al. (2005). Umeda et al. (2005) suggested that the difference in response between the two studies might be due to differences in susceptibility between the two mouse strains, but specific support for this hypothesis is not available.

Investigation of possible liver tumor modes of action is limited to a 13-week dietary study that explored the possible association between biphenyl-induced proliferation of peroxisomes and liver tumors in BDF_1_ mice (Umeda et al. 2004). Evidence of peroxisome proliferation was found in the highest-dose (16,000 ppm) group; however, peroxisome proliferation was not observed at concentrations with increased incidences of liver tumors in the 2-year bioassay in female BDF_1_ mice (Umeda et al. 2005). A peroxisome proliferation-related mode of action could not be established. As biphenyl and its metabolites may have the potential to induce genetic damage, the role of genotoxicity cannot be excluded. Overall, the available data are not adequate to hypothesize a mode of action for biphenyl-induced hepatocarcinogenicity in female mice.

### Human Relevance of Tumor Findings in Experimental Systems

In general, the U.S. EPA takes a public health protective position regarding the interpretation of carcinogenicity data: positive tumor findings in animals in the absence of mode of action information are judged to be relevant to humans (U.S. EPA 2005). In the case of biphenyl, a hypothesized mode of action cannot be developed for liver tumors. Therefore, because there is no evidence indicating that liver tumors in female mice are irrelevant to humans, the tumors are judged relevant. Evaluation of the relevance to humans for urinary bladder tumors in the rat, for which a mode of action has been hypothesized, follows below.

Limited evidence suggests that the proposed mode of action for biphenyl-induced urinary bladder tumors is relevant to humans under the assumption that urinary bladder calculi can be formed in humans at sufficient exposure levels. Calculi resulting from human exposure to substances other than biphenyl have been associated with urinary bladder irritation, regeneration, and cancer (Capen et al. 1999; Cohen 1995, 1998). Four case–control studies of urinary bladder cancer in white human populations published in the 1960s–1980s found relative risks for an association between a history of urinary tract stones and bladder carcinomas ranging from 1.0 to 2.5 (Capen et al. 1999). In addition, a population-based cohort study found a significant excess of bladder cancer in patients hospitalized for kidney or ureter stones [standardized incidence ratio (SIR) of 1.4, 95% confidence interval (CI): 1.3, 1.6]; the risk for women (SIR = 2.8, 95% CI: 2.1, 3.5) was twice that of men (Chow et al. 1997).

Although there is no specific evidence of biphenyl induction of bladder calculi in humans, investigation of biphenyl metabolism *in vitro* using human and rodent tissues reveals that human metabolism of biphenyl is qualitatively similar to rat metabolism [see “Metabolism” section and the Toxicological Review of Biphenyl, Section 3.3 (U.S. EPA 2013) for further details].

Given physiological and anatomical differences between humans and rats, there is uncertainty in extrapolating the dose–response relationship for biphenyl-induced calculi formation in male rats to humans. As discussed earlier, sex-specific differences in calculi formation in the rat suggest that urinary conditions, including urine pH, may play a role in calculi formation. Specifically, the precipitation of 4-HBPOSK in urine to form calculi might be associated with the higher (i.e., more basic) urine pH in male rats exposed to high dietary biphenyl concentrations. Because humans have on average a slightly more acidic urine than the rat (Cohen 1995), humans might be less susceptible than the male rat to the development of urinary bladder calculi.

Another physiological factor potentially contributing to lower susceptibility of humans is the difference in posture between rodents and humans. Based on the anatomy of the urinary tract in humans and their upright, bipedal stature, calculi are either quickly excreted in urine or cause obstruction, leading to pain and subsequent therapeutic removal of the calculi (Cohen 1995, 1998), and thus a relatively shorter duration of irritation of transitional epithelial cells lining the urinary bladder. By contrast, the horizontal quadruped stature of rodents is expected to promote calculi residency time in the bladder without causing obstruction. It is noteworthy that humans hold urine in the bladder and then release the urine at appropriate times, while rodents urinate more often. In humans, only continence under pathological condition, such as neurogenic bladder (i.e., a condition that can cause obstructive bladder), has been associated with increased risk of calculi formation and tumors (Gormley 2010).

### Conclusions Regarding Carcinogenic Hazard

Under the U.S. EPA’s Guidelines for Carcinogen Risk Assessment (U.S. EPA 2005), the database for biphenyl provides “suggestive evidence of carcinogenic potential” based on increased incidence of urinary bladder tumors (transitional cell papillomas or carcinomas) in male F344 rats (Umeda et al. 2002) and liver tumors (hepatocellular adenomas or carcinomas) in female BDF_1_ mice (Umeda et al. 2005) exposed to biphenyl in the diet for 2 years. Selection of this cancer descriptor takes into consideration the fact that urinary bladder tumors appear to be closely related to the formation of urinary bladder calculi occurring in the male rat only at relatively high biphenyl exposure levels.

## Effects Other than Cancer

Epidemiological investigations of possible associations between exposure to biphenyl and health outcomes in humans are limited to two studies of workers exposed to biphenyl during production of biphenyl-impregnated fruit wrapping paper at mills in Finland and Sweden at concentrations above the occupational threshold limit value (1.3 mg/m^3^; ACGIH 2001) (Seppäläinen and Häkkinen 1975; Wastensson et al. 2006). These studies provide limited evidence of nervous system effects (i.e., abnormal electroencephalography and electroneuromyography; increased prevalence of Parkinson’s disease) associated with occupational biphenyl exposure. Experimental animal studies of ingested biphenyl provide consistent evidence that biphenyl exposure is associated with renal toxicity, some evidence that biphenyl exposure may be associated with liver toxicity, and limited evidence that biphenyl exposure may be associated with effects on the urinary bladder and developing fetus (Table 4). The toxicity of inhaled biphenyl has been investigated in two subchronic animal studies (Deichmann et al. 1947; Monsanto 1946; Sun 1977) that provide poor characterization of inhaled biphenyl toxicity because of study limitations that include lack of a control group, high variability in actual chamber concentrations, high mortality due to a malfunction of the temperature control of the inhalation chambers leading to overheating, and limited reporting of study details and histopathological findings. Table 4 summarizes the evidence for biphenyl noncancer toxicity across target organs and systems. Evidence for effects on the kidney, the primary target organ for biphenyl toxicity, is discussed below.

**Table 4:** Evidence for biphenyl noncancer toxicity.

Targets	Key conclusions and evidence
Kidney	Consistent evidence that biphenyl causes renal toxicity Desquamation in both sexes of rats (Umeda et al. 2002) and both sexes of mice (Umeda et al. 2005). Mineralization in both sexes of rats (Umeda et al. 2002) and female mice (Umeda et al. 2005). Necrosis and transitional cell hyperplasia in both sexes of rats (Umeda et al. 2002). Tubular dilation in both sexes of rats (Ambrose et al. 1960; Dow Chemical 1953). Tubular degeneration in male rats (Pecchiai and Saffiotti 1957). Urine BUN level increased in both sexes of mice (Umeda et al. 2005).
Liver	Some evidence that biphenyl causes liver toxicity Increased liver weight in female albino and SD rats, female BDF_1_ mice and monkeys (Ambrose et al. 1960; Dow Chemical 1953; Umeda et al. 2005), but not in male or female F344 rats (Umeda et al. 2002). Histopathological changes and increased liver enzymes were not observed consistently across different species/strains/sexes.
Urinary bladder	Limited evidence that biphenyl causes urinary bladder toxicity Toxicity, including urinary bladder hyperplasia and calculi, was observed in rats (F344 and Wistar) only (Shibata et al. 1989a; Shiraiwa et al. 1989; Umeda et al. 2002). No lesions in urinary bladder in albino or SD rats (Ambrose et al. 1960; Dow Chemical 1953). No changes in the urinary bladder in mice (Imai et al. 1983; Umeda et al. 2005).
Development	Limited evidence that biphenyl causes developmental toxicity A single developmental study found fetal skeletal anomalies (Khera et al. 1979).
Nervous system	Limited evidence that biphenyl causes neurotoxicity Abnormal electroencephalography and electroneuromyography and increases in clinical signs in workers exposed to biphenyl at concentrations that exceeded the occupational limit by up to 100 fold (Seppäläinen and Häkkinen 1975). Increased prevalence of Parkinson’s disease in a factory where exposures were likely to have exceeded the threshold limit value (TLV) of 1.3 mg/m^3^ (Wastensson et al. 2006).

**Figure 1:**
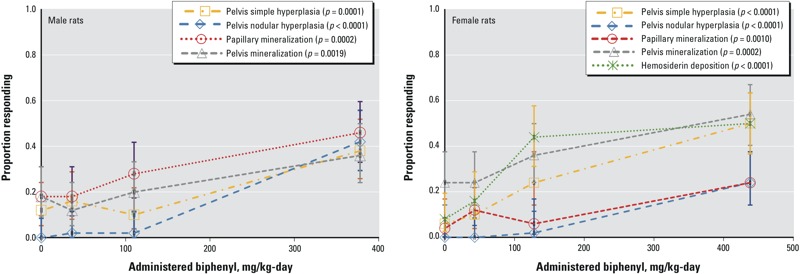
Dose–response relationships for kidney end points in male (left panel) and female (right panel) rats exposed to biphenyl in diet for 2 years. Pelvis hyperplasia (simple and nodular), papillary and pelvis mineralization, and hemosiderin deposition are the most sensitive measures of kidney effects in rats (Umeda et al. 2002). Increased incidences of other histopathological changes (not shown in this graph) in the kidney were observed in high-dose animals only including renal pelvis desquamation, renal pelvis calculi, mineralization of the cortico-medullary junction in male rats; papillary necrosis in male and female rats; and infarct in female rats. Overall, these outcomes support a continuum of kidney effects, increasing in severity with higher exposure. Open symbols represent the observed proportions responding for each outcome, and bars represent corresponding 95% CIs, by exposure group. *p*-Values shown are from one-sided Cochran-Armitage trend tests.

Exposure to biphenyl in the diet for 2 years produced a range of histopathological changes in the kidney of F344 rats (Umeda et al. 2002); Figure 1 depicts the most sensitive, dose-related measures of kidney effects. Mineralization of the papilla (part of the renal medulla) increased in a dose-related manner in both male and female rats; papillary necrosis occurred in both sexes of rats at the high dose only. Papillary mineralization can be found in association with papillary necrosis (Bach and Nguyen 1998), and the histopathologic changes in the medulla overall suggest a continuum of increasing severity of damage from mineralization to necrosis with increasing biphenyl dose. In addition to effects in the papillary region of the medulla, dose-related histopathological changes, including mineralization, transitional cell hyperplasia, desquamation, and calculus formation, were also observed in the renal pelvis of male and female rats (Umeda et al. 2002). Although calculus formation has been observed in the rat kidney (Umeda et al. 2002), the kidney does not appear to be a target of biphenyl carcinogenicity.

A dose-related increase in the incidence of renal hemosiderin deposits was observed in female rats, but not in male rats at any dose level. Hemosiderin is an iron-protein complex that may result from hemoglobin degradation under various conditions (Jennette et al. 2007). However, without information in Umeda et al. (2002) on severity and location of hemosiderin within the kidney, the biological significance of this end point is unclear. Kidney findings were consistently observed in other studies in rats, including tubular dilation or mild tubular degeneration in albino and Sprague-Dawley rats (Ambrose et al. 1960; Dow Chemical 1953; Pecchiai and Saffiotti 1957) and calculi formation in the renal pelvis in Wistar and albino rats (Ambrose et al. 1960; Shiraiwa et al. 1989).

**Figure 2:**
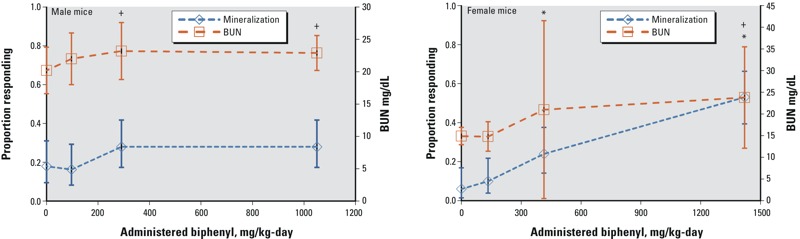
Dose–response relationships for kidney end points in male (left panel) and female (right panel) mice exposed to biphenyl in diet for 2 years. Mineralization in inner stripe-outer medulla and urine BUN are the most sensitive measures of kidney effects in mice (Umeda et al. 2005). Pelvis desquamation, not included in this graph, occurred in high-dose group only. Open symbols represent the observed proportions responding (mineralization) or means (BUN), and bars represent 95% CIs, by exposure group. Note: +, represents statistically significant for BUN by Dunnett’s test (*p* < 0.05); *, represents statistically significant for mineralization by Fisher’s exact test (*p* < 0.05).

In BDF_1_ mice exposed to biphenyl in the diet for 2 years, Umeda et al. (2005) reported dose-related pathological changes in the kidney, including desquamation of the renal pelvis and mineralization of the medulla, and increase in blood urea nitrogen (BUN) levels, the latter evidence of functional disruption of the kidney. The most sensitive measures of kidney toxicity were a dose-related increase in the incidence of mineralization in the inner stripe of outer medulla of the kidney and increased BUN levels, shown in Figure 2. Imai et al. (1983) did not find histopathological changes in the kidney of ddY mice exposed to biphenyl in diet for 2 years. This lack of response in the mouse kidney may be explained by limited power (only ~ 60% of the mice were subjected to pathological examination), and differences in strain susceptibility (Umeda et al. 2005).

In summary, the available evidence supports the finding that kidney toxicity is a potential hazard of biphenyl exposure based on consistent evidence of biphenyl-induced kidney toxicity in studies in rats and some support from studies in mice.

## Discussion

One of the challenges in interpreting the biphenyl health effects data for carcinogencity was that only one of the rat bioassays demonstrated increased incidences of urinary bladder tumors, and that only one of the mouse bioassays demonstrated increased incidences of liver tumors. Systematic consideration of the available bioassays revealed a number of explanations for the seeming lack of agreement within the database for each species.

Given the urinary bladder response demonstrated in the well-conducted 2-year bioassay in rats by Umeda et al. (2002), the other rat bioassays (Ambrose et al. 1960; Dow Chemical 1953; Shiraiwa et al. 1989) were less able to demonstrate a similar tumor response, each for a different set of reasons. The 75-week dietary study in Wistar rats by Shiraiwa et al. (1989) may have been too short to support observation of late-occurring tumors. Small sample sizes for the remaining studies limited the possibility of observing tumors. In Ambrose et al. (1960), evaluation of histopathology only for rats surviving to study termination (as few as two per group at the higher doses) provided an inadequate number of observations to support evaluation of carcinogenic potential. Similarly, the small numbers of Sprague-Dawley rats (12/sex/group) used in the 2-year bioassay by Dow Chemical (1953) and poor survival (including a pneumonia outbreak resulting in deaths of all control male rats by the end of year 1) may have impaired the ability to detect late-developing tumors. All of the studies used a different strain of rat, a known factor associated with sensitivity to cancer (Kacew et al. 1995). Overall, the experimental animal evidence for urinary bladder tumors shows differing, as opposed to conflicting, results.

Mechanistic and toxicokinetic information can bring coherence to disparate findings across species and sexes. As discussed previously, the difference in urinary bladder tumor response in male and female rats is consistent with sex-specific differences in the physical and structural properties of the calculi and with the sex-specific physiological conditions (in particular, urinary pH) that may have led to the differences in calculi properties. The mechanistic basis for species differences in bladder tumor induction by biphenyl has not been investigated. However, as previously discussed, available toxicokinetic data indicate that the amount of 4-HBP, the biphenyl metabolite that plays an essential role in calculi formation, constitutes a larger proportion of total biphenyl metabolites in rats than in mice. The difference in the quantity of 4-HBP formed in rats and mice is concordant with the species differences in bladder tumor response.

In interpreting the biphenyl literature, consideration was also given to evidence from other chemicals that induce urinary bladder tumors. A hypothesized mode of action involving calculi formation, mechanical damage to transitional epithelium, regenerative proliferation, subsequent hyperplasia, and urinary bladder tumor induction has been proposed for other chemicals, including melamine, uracil, and the sodium salt of 2-HBP, that all induce urinary bladder tumors in rodents (Capen et al. 1999; Cohen 1995, 1998; IARC 1999). These findings provide precedent for the proposed mode of action of biphenyl, which follows a similar series of key events in male rats.

Similar to the urinary bladder tumor findings, the evidence for liver tumor responses associated with biphenyl exposure generally shows differing, rather than conflicting, results, given the variety of study designs used. The positive liver tumor finding in female BDF_1_ mice in the well-conducted 2-year study by Umeda et al. (2005) was not observed in any other mouse studies. NCI (1968) found no liver tumor response in B6C3F_1_ or B6AKF_1_ mice, but these evaluations were carried out at a lower dose than those used by Umeda et al. (2005), for a shorter duration (18 rather than 24 months), and with treated groups of no more than 18 animals. Imai et al. (1983) did not observe an increased incidence of liver tumors in a 2-year bioassay in ddY mice at a dose that induced liver tumors in female BDF_1_ mice. The reason for the difference in response between Imai et al. (1983) and Umeda et al. (2005) is unknown, but may be related to mouse strain differences (Umeda et al. 2005). Unlike biphenyl-induced urinary bladder tumors, the limited mechanistic and toxicokinetic information related to liver tumor formation provides little insight into the different liver tumor responses across sexes of mice.

As noted in U.S. EPA (2005), support for human carcinogenic potential of an agent is provided by positive results in animal experiments in more than one species, sex, or site. For biphenyl, the two positive cancer results, bladder tumors and liver tumors, are in different sexes of different species (male rats and female mice, respectively). Support for carcinogenic potential would be increased if at least one of these species–sex groups had demonstrated multiple tumor types, such as both of these outcomes. The fact that neither site was repeated in the other group is unremarkable, however, because site concordance across species or sexes is not always expected (U.S. EPA 2005). Finally, although replication of the observed outcomes in similar studies would provide greater confidence in this human carcinogenic potential determination, strong support follows from the outcomes being demonstrated in the best quality studies available, namely, Umeda et al. (2002) and Umeda et al. (2005); both studies used a relatively low range of dose levels and evaluated a larger number of animals (50/sex/exposure group) among the available studies, and were conducted in the same laboratory, increasing comparability across the rat and mice results.
